# Epidemiology and risk factors for nosocomial infection in the respiratory intensive care unit of a teaching hospital in China: A prospective surveillance during 2013 and 2015

**DOI:** 10.1186/s12879-019-3772-2

**Published:** 2019-02-12

**Authors:** Linchuan Wang, Kai-Ha Zhou, Wei Chen, Yan Yu, Si-Fang Feng

**Affiliations:** 1 Department of Clinical Laboratory of The First Affiliated Hospital of Xi’an Jiaotong University, Xi’an, Shaanxi Province China; 2 Department of Clinical Laboratory of Hospital of Xi’an Jiaotong University, Xi’an, Shaanxi Province China; 3 Department of Clinical Laboratory of Honghui Hospital, Xi’an JiaotongUniversity, Xi’an, Shaanxi Province China; 4 Department of Respiratory Intensive Care Unit of The First Affiliated Hospital of Xi’an Jiaotong University, Xi’an, Shaanxi Province China

**Keywords:** Nosocomial infection, Respiratory intensive care unit, Risk factors

## Abstract

**Background:**

To determine the epidemiology and risk factors for nosocomial infection (NI) in the Respiratory Intensive Care Unit (RICU) of a teaching hospital in Northwest China.

**Methods:**

An observational, prospective surveillance was conducted in the RICU from 2013 to 2015. The overall infection rate, distribution of infection sites, device-associated infections and pathogen in the RICU were investigated. Then, the logistic regression analysis was used to test the risk factors for RICU infection.

**Results:**

In this study, 102 out of 1347 patients experienced NI. Among them, 87 were device-associated infection. The overall prevalence of NI was 7.57% with varied rates from 7.19 to 7.73% over the 3 years. The lower respiratory tract (43.1%), urinary tract (26.5%) and bloodstream (20.6%) infections accounted for the majority of infections. The device-associated infection rates of urinary catheter, central catheter and ventilator were 9.8, 7.4 and 7.4 per 1000 days, respectively.The most frequently isolated pathogens were *Staphylococcus aureus* (20.9%)*, Klebsiella pneumoniae* (16.4%) and *Pseudomonas aeruginosa* (10.7%). Multivariate analysis showed that the categories D or E of Average Severity of Illness Score (ASIS), length of stay (10–30, 30–60, ≥60 days), immunosuppressive therapy and ventilator use are the independent risk factors for RICU infection with an adjusted odds ratio (OR) of 1.65 (95% CI: 1.15~2.37), 5.22 (95% CI: 2.63~10.38)), 2.32 (95% CI: 1.19~4.65), 8.93 (95% CI: 3.17~21.23), 31.25 (95% CI: 11.80~63.65)) and 2.70 (95% CI: 1.33~5.35), respectively.

**Conclusion:**

A relatively low and stable rate of NI was observed in our RICU through year 2013–2015. The ASIS-D、E, stay ≥10 days, immunosuppressive therapy and ventilator use are the independent risk factors for RICU infection.

## Background

Nosocomial infection (NI) which also called “hospital-acquired or health care-associated infection” is a serious public health issue affecting hundreds of millions of people every year worldwide [1]**.** NI is defined as an infection occurring in a patient admitted to the health-care settings for more than 48 but without any evidence that the infection was present or incubating at the time of admission [1–3]**.** In the hospitals or other health care facilities, NI is a leading cause of increased morbidity, mortality and financial burden [1–7]**.** The incidence of NI as most studies reporting data ranged from 3.6 to 12% in high-income countries [8–10] and 5.7 to 19.1% in low- and middle-income coutries [8, 11]**.** Predisposing factors, i.e., the invasive procedures [12–15], long hospital stay [16], excessive antibiotics usage [9] and the existence of severe illness [17] lead to NI rate in patients admitted to the intensive care unit (ICU) several fold higher than that in the general hospital population [18–21]**.** Now, NI is more concerned as the focus of safety and quality improvements efforts in many hospitals. The study was designed to investigate the epidemiology, risk factors and outcome of NI in a Respiratory ICU (RICU) at the largest teaching hospital in Northwest China.

## Methods

### Study population

This study was conducted in RICU of the First Affiliated Hospital of Xi’an Jiaotong University, which is the largest hospital in Northwest China. It is a 2541-bed teaching hospital with a 16-bed RICU and about 3 millions outpatients annually. The nurse-to-patient ratio in RICU is about 1: 2–3 per shift. A total of 1347 patients admitted to the RICU for more than 48 h were included in the study from January 2013 to December 2015. NI was defined as an infection developed after 48 h of RICU admission and diagnosed according to the the American Center for Disease Control and Prevention (CDC) criteria [22]**.** In the study, the infection on a different site and with different pathogens from the primary infection that occurred at least 48 h after admission to the RICU was also classified as NI.

### Data collection

The patients were followed until discharge from RICU or death, and the information on each patient was recorded on the standard surveillance paper chart. All patients with suspected infection underwent liver and renal function test, whole blood count、urine、fecal and coagulation profile examinations, chest radiography, blood、tracheal aspirate and other body fluids cultures as clinically indicated. Demographic information, i.e., the gender, age, admission and discharge dates, temperature, admission diagnosis, comorbidity, device use and the period of application, laboratory tests, chest radiographs, the isolated pathogens and susceptibility testing to antimicrobial agents, infection sites, drug usage were collected.

#### The assessment of ASIS

The disease severity was assessed by the Average Severity of Illness Score (ASIS), which was from the Standard for Nosocomial Infection Surveillance of China and established by China Ministry of Health. The criteria of ASIS was as follows: **ASIS-A**: The patients should be required only routine monitor without intensive care and treatment, and they usually discharged from ICU within 48 h; **ASIS-B**: The patients, such as the cases admitted to ICU to exclude myocarditis or myocardial infarction, were in stable condition and just required preventive monitor without intensive care and treatment; **ASIS-C**: The patients, such as those with chronic renal failure, were in stable condition and required intensive care; **ASIS-D**: The patients in unstable condition but without coma, shock and Disseminated Intravascular Coagulation (DIC), should be performed intensive care and treatment. The treatment should be regularly evaluated and adjusted; **ASIS-E**: The patients with unstable condition were in coma or shock. The cardio-pulmonary resuscitation, intensive care and treatment should be performed. The intensive care and treatment should be regularly evaluated and adjusted.

According to the Standard for Nosocomial Infection Surveillance of China, the gender, age, admission diagnosis, disease severity, comorbidity, immunosuppressive therapy and invasive procedures were investigated as the potential risk factors for NI in the study.

### Research indexes and definitions

The prevalence of nosocomial infection rate was calculated by dividing the total number of nosocomial infections by the total number of patients (× 100). The device-associated nosocomial infection rate was calculated by dividing the total number of device-associated infection by the total days of device application (× 1000). The device utilization (DU) ratios was calculated by dividing the days of device application by the total patient days.

### Statistical analysis

Statistical analyses were performed using SPSS 13.0 (serial number 5026743; SPSS Inc., Chicago, IL, USA). Descriptive frequencies were expressed using mean (standard deviation). Chi-square tests were used to compare the rates. For evaluating risk factors of NI, univariate analysis and multivariable logistic regression analysis were used to derive crude OR and adjusted OR, respectively. A *p*-value < 0.05 was considered statistically significant.

## Results

### RICU admission patients’ characteristics, demographic and clinical data

**Fig. 1:**
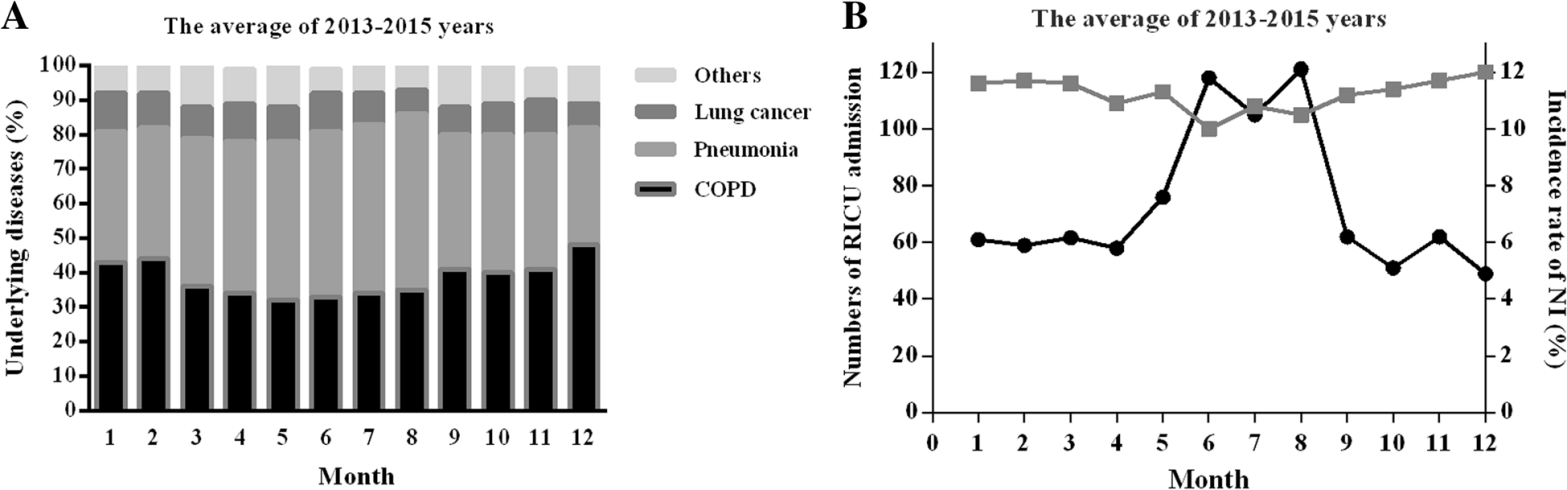
The distributions in each month of **a** RICU admission diagnosis, **b** patients admitted to the RICU and incidence rate of NI

During the study period, a total of 1347 patients were included, 893 males (66.3%) and 454 females (33.7%), with a mean age of 58.6 years (SD = 17.1). The average length of RICU stay was 8.54 ± 17.72 days, giving 11,501 patient-days. The pneumonia, chronic obstructive pulmonary disease (COPD) and lung cancer accounted for the majority of the RICU admission diagnosis (40.98, 38.9 and 11.6%, respectively). According with ASIS, the patients were mainly in B (42.69%) and C (33.78%) grades. The patients distribution in each month during 2013–2015 was no significant difference (one-way ANVOA, *p* = 0.064) with 112.2 ± 7.5 numbers per month, the highest and lowest numbers were observed in December (120) and June (100), respectively, Fig. 1a. The COPD exacerbated in December, January and February, pneumonia (community acquired pneumonia) more appeared in July and August, but the proportion of lung cancer in each month was close Fig. 1a. The characteristics of the RICU admission patients were shown in Table 1.

**Table 1:** The characteristics of 1347 patients admitted to the RICU

Parameter	Overall (*n* = 1347)		Incidence of nosocomial infection			
	No	% /$\overline {\mathrm{X}}$± s	No	%	χ^2^	*p*- value
Age, years		58.6 ± 17.1				
Gender						
Male	893	66.3	67	7.50	0.016	0.9
Female	454	33.7	35	7.71		
Admission diagnosis						
COPD	524	38.90	31	5.92	8.438	0.038
Pneumonia	552	40.98	43	7.79		
Lung cancer	156	11.58	21	13.46		
Others	115	8.54	7	6.09		
ASIS class						
A	221	16.41	9	4.07	49.42	< 0.001
B	575	42.69	27	4.7		
C	455	33.78	45	9.89		
D	73	5.42	13	17.81		
E	23	1.71	8	34.78		
Years						
2013	431	32.00	31	7.19	0.213	0.795
2014	450	33.41	35	7.78		
2015	466	34.60	36	7.73		
RICU stay, days						
<10	775	57.54	19	2.45	134.998	0.000
10~30	445	33.04	33	7.42		
30~60	72	5.35	21	29.2		
≥ 60	55	4.08	29	52.7		

### The characteristics of of nosocomial infection in RICU

**Fig. 2:**
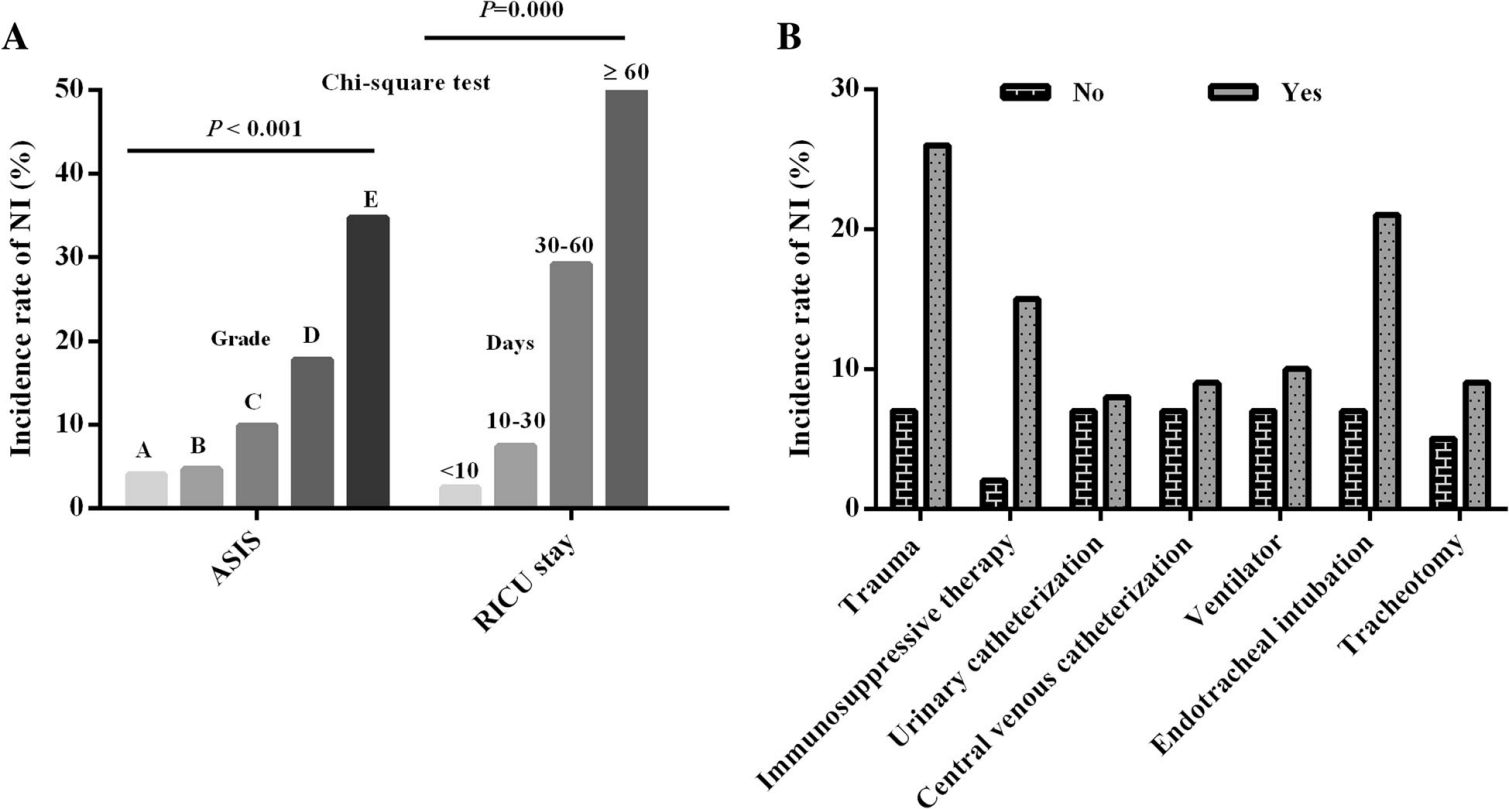
The comparison of incidence rate of NI for **a** patients with different ASIS grades and length of RICU-stay, **b** patients with presence or absence of immunosuppressive therapy, endotracheal intubation, tracheotomy, urinary catheterization, central venous catheterization and ventilator

During the study, 43 of the 552 cases admitted to the RICU with community acquired pneumonia developed NI (a different pathogens than the initial one was isolated). In total, 102 out of 1347 patients experienced NI, 67 males and 35 females, with a prevalence of 7.57% (8.9 per 1000 days). The incidence rate of NI in male (7.5%) was close to that in female (7.7%), *p* = 0.90. There is no significant change in the incidence rate of NI during the 3 years (range: 7.19 to 7.73%), *p* = 0.795. The NI in RICU occurred frequently in June, July and August, Fig. 1b. The NI rate in patients with lung cancer (13.5%) was significantly higher than that in patients with pneumonia (7.9%) and in patients with COPD (6.1%), *p* = 0.038. With the severity of disease progression from A to E grade, the NI rate increased from 4.07 to 34.78%, *p* < 0.001, Fig. 2a. The increasing of NI was also found when the length of RICU stay prolonged, *p* = 0.000, Table 1, Fig. 2b.

One hundred seventy-seven pathogens were isolated and identified from the 102 infections, 83 g-negative bacilli and 63 g-positive cocci and 31 fungi. *Staphylococcus aureus* (20.9%)*, Klebsiella pneumoniae* (16.4%) and *Pseudomonas aeruginosa* (10.7%) were the most frequently isolated pathogens. The lower respiratory tract, urinary tract and bloodstream accounted for the majority of the RICU-acquired infections (43.1, 26.5 and 20.6%, respectively), Table 2.

**Table 2:** The infection sites and pathogens isolated in nosocomial infections

Causative organism	No	%
*Gram*-negative bacilli (*n* = 83)		
*Klebsiella pneumoniae*	29	16.4
*Pseudomonas aeruginosa*	19	10.7
*Escherichia coli*	17	9.6
*Acinetobacter baumanii*	10	5.6
*Pseudomonas cepacia*	5	2.8
Others	3	1.8
*Gram*-positive cocci (*n* = 63)		
*Staphylococcus aureus*	37	20.9
*Stahylococcus epidermidis*	16	9.0
*Streptococcus viridans*	6	3.4
Others	4	2.3
*Fungi* (*n* = 31)		
*Candida albicans*	15	8.5
*Candida parapsilosis*	5	6.2
*Aspergillus*	11	2.8
Total (overall)	177	100.0
Infection sites	No	%
Lower respiratory tract	44	43.1
Upper respiratory tract	2	26.5
Urinary tract	27	20.6
Blood stream	21	4.9
Gastrointestinal tract	5	2.9
Surgical sites	3	2.0
Total (overall)	102	100.0

### Device-associated nosocomial infection in RICU

A total of 87 device-associated nosocomial infections, i.e., 28 catheter-associated urinary tract infections (CAUTI), 12 catheter-associated bloodstream infections (CABSI) and 47 ventilator-associated pneumonia (VAP) were detected in 1347 patients, resulting in an overall rate of 6.5% (7.6 per 1000 days) and accounting for 85.3% of RICU-acquired infections. During the study period, the device application was 3767 days for urinary catheter, 1615 days for central catheter and 4804 days for ventilator, with a device utilization ratio of 0.33, 0.14 and 0.42, respectively. The rate of infection was 9.8 per 1000 days of VAP, 7.4 per 1000 days of CAUTI and 7.4 per 1000 days of CABSI, Table 3. The correlation coefficients between the device utilization and NI were 0.41 for urinary catheter (*p* = 0.017), 0.139 for central catheter (*p* = 0.087) and 0.314 for ventilator (*p* = 0.003). No significant differences were observed between the VAP,

**Table 3:** The device-associated infection rate and device utilization (DU) ratio

Month	Patient days	CAUTI				CABSI				VAP			
		No	Catheter days	CAUTI rate	DU ratio	No	Catheter days	CABSI rate	DU ratio	No	Ventilator days	Vap rate	DU ratio
Jan	1161	3	437	6.9	37.6	0	152	0	13.1	5	474	10.6	40.8
Feb	972	3	314	9.6	32.3	1	74	13.4	7.6	4	381	10.5	39.2
Mar	1100	4	360	11.1	32.7	1	152	6.6	13.8	3	566	5.3	51.5
Apr	1045	1	243	4.1	23.3	0	198	0	18.9	5	474	10.5	45.4
May	947	3	210	14.3	22.2	1	25	40.3	2.6	3	482	6.2	50.9
Jun	769	3	205	14.6	26.7	1	118	8.5	15.3	3	335	9	43.6
Jul	853	3	248	12.1	29.1	3	130	23	15.2	6	304	19.7	35.6
Aug	748	2	197	10.2	26.3	1	37	26.9	4.9	7	262	26.7	35.0
Sep	805	3	464	6.5	57.6	1	153	6.5	19.0	4	253	15.8	31.4
Oct	1083	0	451	0	41.6	1	226	4.4	20.9	2	492	4.1	45.4
Nov	970	1	344	2.9	35.5	0	192	0	19.8	3	478	6.3	49.3
Dec	1048	2	311	6.4	29.7	2	164	12.2	15.6	2	295	6.8	28.1
Total	11,501	28	3784	7.4	32.9	12	1622	7.4	14.1	47	4796	9.8	41.7

CAUTI and CABSI rates (*χ*^*2*^ = 0.412, *P* = 0.810).

### Risk factors analysis for nosocomial infection in RICU

There are 16 potential risk factors for NI in RICU (Table 4). In the univariate analysis, underlying diseases (lung cancer), ASIS-C˴ D˴ E, RICU stay (≥ 10 days), trauma, diabetes mellitus, immunosuppressive therapy, endotracheal intubation, tracheotomy, utilization of urinary catheter, central catheter and ventilator were identified as risk factors for NI in RICU, *P <* 0.05.

**Table 4:** The risk factors for nosocomial infection in RICU

Factors	No		Crude			Adjusted		
	Patients with infections	Patients without infections	OR	95%CI	p-value	OR	95%CI	p-value
Age, years								
< 60	35	826	1			1		
≥ 60	67	419	0.97	0.64~1.49	0.892	1.43	0.81~2.55	0.221
Gender								
Male	67	826	1			1		
Female	35	419	0.97	0.64~1.49	0.892	0.79	0.44~1.41	0.423
Admission diagnosis								
COPD	31	493	1			1		
Pneumonia	43	509	1.34	0.83~2.17	0.226	0.16	0.02~1.26	0.082
Lung cancer	21	135	2.47	1.38~4.44	0.002	0.11	0.02~0.80	0.059
Others	7	108	1.03	0.44~2.40	0.944	0.18	0.03~1.20	0.076
ASIS								
A	9	212	1			1		
B	27	548	1.16	0.54~2.51	0.705	1.16	0.81~1.66	0.412
C	45	410	2.59	1.24~5.39	0.011	1.44	0.92~2.25	0.116
D	13	60	5.10	2.08~12.52	0.000	1.65	1.15~2.37	0.007
E	8	15	12.56	4.24~37.25	0.000	5.22	2.63~10.38	0.000
RICU stay, days								
*<*10	19	756	1			1		
10~30	33	412	3.19	1.79~5.48	0.000	2.32	1.19~4.65	0.018
30~60	21	51	16.38	8.28~32.41	0.000	8.93	3.17~21.23	0.000
≥ 60	29	26	44.38	22.08~89.21	0.000	31.25	11.80~63.65	0.000
Diabetes mellitus								
No	35	795	1			1		
Yes	67	450	3.38	2.21~5.17	0.000	1.14	0.94~1.38	0.183
Hypertension								
No	66	889	1			1		
Yes	36	356	1.36	0.89~2.08	0.153	1.06	0.88~1.26	0.321
Cerebrovascular diseases								
No	71	895	1			1		
Yes	31	350	1.12	0.72~1.73	0.623	1.05	0.81~1.24	0.226
Post-operative tumor								
No	82	1063	1			1		
Yes	20	182	1.43	0.85~2.38	0.177	1.06	0.87~1.15	0.197
Trauma								
No	82	1187	1			1		
Yes	20	58	4.99	2.87~8.70	0.000	1.23	0.92~1.27	0.08
Immunosuppressive therapy								
No	13	729	1			1		
Yes	89	516	9.67	5.35~17.50	0.000	1.82	1.53~4.06	0.013
Urinary catheterization								
No	39	533	1			1		
Yes	63	712	1.21	0.80~1.83	0.369	1.27	0.94~1.71	0.116
Central venous catheterization								
No	55	753	1			1		
Yes	47	492	1.31	0.87~1.96	0.195	1.30	0.78~2.17	0.318
Ventilator								
No	78	1027	1			1		
Yes	24	218	1.45	0.90~2.34	0.13	2.70	1.33~5.35	0.006
Endotracheal intubation								
No	83	1172	1			1		
Yes	19	73	3.68	2.12~6.38	0.000	1.28	0.81~2.06	0.283
Tracheotomy								
No	23	467	1			1		
Yes	79	778	1.67	1.03~2.69	0.036	1.14	0.78~1.52	0.389

**Fig. 3:**
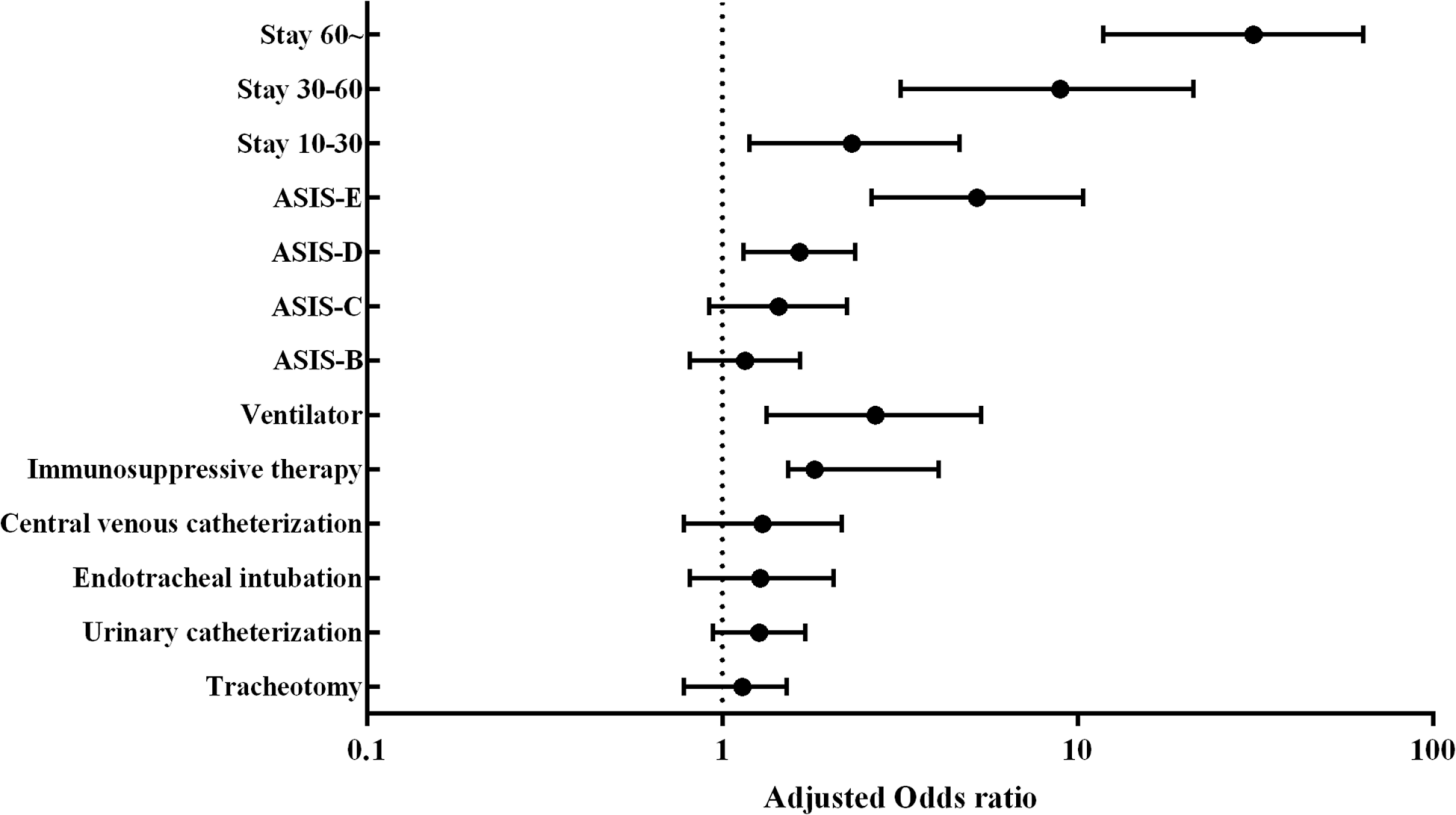
The adjusted odds ratio and 95% confidence intervals of risk factors for RICU infection by multivariate analysis

Multivariable logistic regression analysis was conducted to control for the effects of confounding variables. The final analysis showed that ASIS-D˴E, RICU stay (≥ 10 days), immunosuppressive therapy and ventilator utilization are independent risk factors. In RICU ward, patients who were in D˴ E grade, with immunosuppressive therapy, 10–30˴ 30–60 and ≥ 60 days stay and ventilator utilization were 1.65, 5.22, 1.82, 2.32, 8.93, 31.25 and 2.70 times, respectively, more likely to develop NI compared to the control patients who were in A grade, absence of immunosuppressive therapy, with < 10 days stay, and absence of ventilator utilization, respectively, Table 4, Fig. 3. One hundred forty-six patients died during the study period, 21 patients with NI and 125 patients without NI, with a mortality rate of 10.8% (12.7 per 1000 days). The mortality rate in patients with NI was 20.6%, which was significantly higher than that in patients without NI (10.4%), *p* = 0.001. The incidence of death in patients with NI was 2.32 times to those without NI (95% CI: 1.39–3.89).

## Discussion

NI causes increased morbidity, mortality and financial burden at the hospital setting [1–7, 23]**.** The infection surveillance and risk factors analysis are important prerequisites for the prevention and treatment of NI. At present, abundant literatures focus on the healthcare-associated infection [4, 6, 9–11, 17], infection in ICU [16, 18–21, 23] and device-associated infection [12–15] have been reported. However, few studies on the topic of infection in RICU have been published. Thus, we conducted this prospective surveillance during 2013 and 2015 to determine the epidemiology and risk factors for NI in RICU at the First Affiliated Hospital of Xi’an Jiaotong University, China. But it was a single cente study and from the largest hospital in Northwest China. The selective bias of the study may affect the generalization of the results.

In our study, there was no significant change in the incidence rate of NI over the 3 years. The overall prevalence of NI in RICU was 7.57%, which was lower than the published rates in European survey (8%) [24] and in India (33.5%) [25]. The mean length of stay was 8.54 days, which was lower than that reported in Italy [26]**.** In our RICU, COPD was the common underlying diseases, which is in agreement with the published study [26]**.** Similar to previous reports from other countries,^**24, 25**^ the most frequently isolated pathogens were *Staphylococcus aureus, Klebsiella pneumoniae* and *Pseudomonas aeruginosa*. The common distribution of RICU infections were lower respiratory tract, urinary tract and bloodstream, this is similar to the reports for ICU infection in China [15], European [9, 16] and Malaysian [27]**.**

In the present study, the device-associated infection accounted for the most of RICU-acquired infections (85.3%). The device utilization ratios (0.14–0.42) were lower than the published rates in Europe, Malaysian and surveys from 61 countries (0.52–0.95) [12, 14, 27–29]**.** The VAP rate in our study was significantly lower than that in Greece [14], Malaysian [27]and surveys from 61 countries [28, 29] where the rates varied from 13.6 to 20 per 1000 days. The CAUTI rate in our study was lower than that in Malaysian (15.6 per 1000 days) [27], but higher than the published rates (4.2–6.3 per 1000 days) [14, 28, 29]**.** The CABSI rate in the present study was lower than that in Greece (11.8 per 1000 days) [14], but higher than that in Malaysian (3.0 per 1000 days) [27]**.**

Previous studies [11, 14, 16, 17, 25, 30] indicated that surgery, device utilization, antimicrobial use and length of stay were the risk factors for NI. In our study, the incidence of RICU infection in patients with stay (≥ 10 days), ASIS-C˴ D˴ E, lung cancer, trauma, diabetes mellitus, immunosuppressive therapy, tracheotomy, device utilization was significant higher than that in the control patients (*P <* 0.05). But only ASIS-D˴ E, stay ≥10 days, immunosuppressive therapy and ventilator utilization are independent risk factors for RICU infection (*P <* 0.05). The incidence of death in patients with NI was 2.32 times to those without NI.

## Conclusions

In conclusion, a relatively low and stable rate of NI was observed in our RICU through year 2013–2015. ASIS-D˴ E, stay ≥10 days, immunosuppressive therapy and ventilator use are independent risk factors for developing infection in our RICU. High mortality rates in patients with infection suggest that infection control activities in RICU must be constantly maintained in order to reduce the rate.

## Abbreviations

ASIS: Average severity of illness score
CABSI: Catheter-associated bloodstream infections
CAUTI: Catheter-associated urinary tract infections
CDC: Center for disease control and prevention
COPD: Chronic obstructive pulmonary disease
DIC: Disseminated intravascular coagulation
DU: Device utilization
ICU: Intensive care unit
NI: Nosocomial infection
OR: Odds ratio
RICU: Respiratory intensive care unit
VAP: Ventilator-associated pneumonia
